# Identification of inhibitors of the transmembrane protease FlaK of *Methanococcus maripaludis*

**DOI:** 10.1002/mbo3.358

**Published:** 2016-04-01

**Authors:** Ina Coburger, Yvonne Schaub, Dirk Roeser, Kornelia Hardes, Patrick Maeder, Nina Klee, Torsten Steinmetzer, Diana Imhof, Wibke E. Diederich, Manuel E. Than

**Affiliations:** ^1^ Leibniz Institute on Aging (FLI)Protein Crystallography GroupBeutenbergstr. 11Jena07745Germany; ^2^ Department of Pharmaceutical ChemistryPhilipps University MarburgMarbacher Weg 6Marburg35032Germany; ^3^ Institute of PharmacyPharmaceutical Chemistry IUniversity of BonnBrühler Str. 7Bonn53119Germany

**Keywords:** Gel‐shift protease assay, I‐CLiP, inhibitors, membrane proteins, TFPP.

## Abstract

GxGD‐type intramembrane cleaving proteases (I‐CLiPs) form a family of proteolytic enzymes that feature an aspartate‐based catalytic mechanism. Yet, they structurally and functionally largely differ from the classical pepsin‐like aspartic proteases. Among them are the archaeal enzyme FlaK, processing its substrate FlaB2 during the formation of flagella and *γ*‐secretase, which is centrally involved in the etiology of the neurodegenerative Alzheimer's disease. We developed an optimized activity assay for FlaK and based on screening of a small in‐house library and chemical synthesis, we identified compound 9 as the first inhibitor of this enzyme. Our results show that this intramembrane protease differs from classical pepsin‐like aspartic proteases and give insights into the substrate recognition of this enzyme. By providing the needed tools to further study the enzymatic cycle of FlaK, our results also enable further studies towards a functional understanding of other GxGD‐type I‐CLiPs.

## Introduction

Archaea as well as bacteria possess flagella that are important for their movement in water and on surfaces. Although similar in function, there are major differences between the flagella of both kingdoms of life. Flagella of archaea consist of multiple subunits and are expressed as precursor proteins, the so‐called preflagellins, containing a signal sequence at their N–terminus (Kalmokoff et al. 1990; Albers et al. 2006; Trachtenberg and Cohen‐Krausz 2006). Interestingly, sequence alignments demonstrated that this region is homologous to the type IV prepilins of bacteria (Faguy et al. 1994; Bardy and Jarrell 2002). The biological function of the latter proteins is different, however, as they play an important role for genetic gene transfer, virulence, surface attachment, and twitching motility (Dubnau 1997; Fernandez and Berenguer 2000; Lapointe and Taylor 2000). This sequence stretch of both, bacterial type IV prepilins and archaeal preflagellins, consists of a short positively charged peptide, followed by a protease cleavage site and a hydrophobic transmembrane helix (Ng et al. 2007). Based on this discovery, it was suggested that also archaea possess proteases cleaving these signal sequences (Albers et al. 2003) that are similar to the bacterial type IV prepilin peptidases (TFPPs) (Lory and Strom 1997). Indeed, preflagellin processing peptidases could be identified. For the membrane‐bound protease FlaK of *Methanococcus maripaludis* (Bardy and Jarrell 2002) and *Methanococcus voltae* (Bardy and Jarrell 2003), preflagellin peptidase activity could be demonstrated. Hereby, these proteases cleave the so‐called FlaB proteins. Mutations of the *flaK* gene, leading to inactivation of the enzyme, prevent formation of flagella and thus hamper motility (Bardy and Jarrell 2003). *M. maripaludis* contains different substrates for FlaK (FlaB1, FlaB2, and FlaB3) important for the generation of the archaeal flagella (Bardy et al. 2003). The signal peptide of FlaB2 consisting of 12 amino acids is cleaved after a conserved glycine (Fig. S1).

Similar to the TFPPs, also FlaK contains two aspartyl residues, which are essential for the proteolytic processing of its substrates. Both aspartates are localized on the cytoplasmic side of the membrane (Ng et al. 2007; Hu et al. 2011). Mutagenesis of these aspartates leads to the inactivation of FlaK (Bardy and Jarrell 2003). Accordingly, it is generally accepted that FlaK is an aspartic protease. FlaK does, however, not contain the classical D‐T/S‐G motif of prototypical aspartic proteases, but shows the conserved GxGD motif that is also found in TFPPs and presenilin (Steiner and Haass 2000). Also, the pH optimum of FlaK is in the neutral range, another similarity to the TFPPs (Bardy and Jarrell 2003). Based on these findings, it was suggested that FlaK and TFPPs might have a similar reaction mechanism and that both proteins are homologous regarding the structure of their active sites (Ng et al. 2006). Due to their similarity to presenilin, both enzymes are also often considered as model proteases for *γ*‐secretase of rather simple transmembrane architecture. The large transmembrane complex *γ*‐secretase consists of the four subunits (or components) – presenilin, nicastrin, anterior pharynx‐defective 1 and presenilin enhancer 2 (Edbauer et al. 2003; Li et al. 2009), and proteolytically liberates the amyloidogenic peptide A*β* during the etiology of the neurodegenerative Alzheimer's disease (AD) (Zhang et al. 2012). Although a recent determination of the structure of *γ*‐secretase by cryo‐EM single‐particle analysis (Lu et al. 2014; Bai et al. 2015) gave insight into the spatial arrangement of the four component proteins, an highly resolved atomic picture of its active site as required for the detailed analysis of its reaction mechanism and the structure‐based development of protease inhibitors remains to be established. Thus, any advance in our understanding of the catalytic mechanism of FlaK and its inhibition would inevitably also largely increase our knowledge about *γ*‐secretase and its functionality in AD. In addition, it is known that inhibitors often stabilize enzymes in crystallographic studies by locking them into a catalytically relevant conformation. The development of a respective FlaK‐inhibitor would thus not only represent a way to interfere with its activity but also support its crystallization in a catalytically competent state and help to understand the active site organization and functionality of all GxGD‐family intramembrane cleaving proteases (I‐CLiPs).

Recently, the structure of FlaK from *M. maripaludis* was solved by protein crystallography (Hu et al. 2011). It was demonstrated that FlaK consists of two compactly folded domains, the *α*‐helical membrane spanning domain with six transmembrane helices and a solvent exposed domain with four antiparallel *β*‐strands. Compared to the prototypical aspartic protease, pepsin confirmed a fundamentally different architecture. FlaK seems to be rather similar to the I‐CLiP presenilin, for which a structural model was deduced from cysteine‐scanning mutagenesis and cross‐linking experiments (Sato et al. 2006, 2008; Tolia et al. 2006; Hu et al. 2011) as well as its EM‐structure (Lu et al. 2014; Bai et al. 2015). A 12 Å gap does, however, separate the catalytically important aspartates in the FlaK structure resulting in an inactive conformation (PDB‐ID: 3S0X) (Hu et al. 2011). In addition, the crystal structure of another signal peptide peptidase and presenilin/SPP homolog (PSH) from the archaeon *Methanoculleus marisnigri* JR1 has recently been solved. It does, however, also show an inactive conformation (Li et al. 2013). The two catalytically important aspartates are herein separated by 6.7 Å, suggesting that substrate binding may trigger a conformational change. A more recent study by the same lab (Dang et al. 2015) shows binding of a *γ*‐secretase inhibitor to PSH. Nevertheless, the details of catalysis and inhibitor interaction are still largely open. Correspondingly, the details of the reaction mechanism of FlaK and other I‐CLiPs still remain to be characterized in more detail.

We herein describe the development of an optimized activity assay for FlaK of *M. maripaludis,* which laid the foundation to screen a small in‐house library of structurally diverse aspartic protease inhibitors. Our identification of the first inhibitors of FlaK as well as their further chemical optimization and analysis resulted in the nonpeptidic compound **9**, being an invaluable tool to obtain deeper insights into the reaction mechanism of this family of aspartic proteases.

## Materials and Methods

### In vivo activity assay

To analyze whether FlaK is actively expressed in *E. coli*, Tuner (DE3) cells were transformed with pIK2‐flaB2 (encoding FlaB2 C‐terminally fused to a hexa‐Histidine and HA‐tag, HA‐tag = amino acids 98–106 of Human influenza hemagglutinin) and p948 (encoding FlaK C‐terminally fused to a hexa‐Histidine tag, personal gift of S.‐V. Albers) and grown in lysogeny broth (LB) medium at 37°C. Expression of both proteins was induced by the addition of 1 mmol/L isopropyl‐β‐D‐thiogalactopyranosid (IPTG). After 4 h, cells were harvested, resuspended in 50 mmol/L Tris•HCl pH 7.4, 150 mmol/L NaCl, and samples were analyzed by western blot using the anti‐HA antibody.

### Expression and purification of FlaK

*E. coli* Tuner (*DE3*) cells carrying p948 were grown in LB medium at 37°C to mid‐log growth phase and expression was induced by the addition of 1 mmol/L IPTG. Cells were harvested 4 h postinduction and resuspended in 20 mM Tris•HCl pH 7.4, 150 mM NaCl, 5% glycerol. Following cell disruption using a 110‐S Microfluidizer equipped with a H10Z interaction chamber (Microfluidics), unbroken cells and inclusion bodies were removed by centrifugation at 4000 g for 20 min. Membranes were collected by ultracentrifugation at 100,000 g for 1 h and resuspended in 20 mmol/L Tris•HCl, 150 mmol/L NaCl, 5% glycerol, pH 7.4. Following solubilization with 1% n‐dodecyl‐β‐D‐maltopyranoside (DDM) at 4°C overnight and another ultracentrifugation step at 100,000 g for 1 h, the supernatant containing solubilized FlaK was diluted 1:2 with 20 mmol/L Tris•HCl pH 7.4, 150 mmol/L NaCl, 5% glycerol and loaded onto a HisTrap FF Crude affinity column (GE Healthcare). The column was washed with 20 mmol/L Tris•HCl pH 7.4, 150 mmol/L NaCl, 5% glycerol, 0.02% DDM, 35 mmol/L imidazole, and FlaK was eluted with 20 mmol/L Tris•HCl pH 7.4, 150 mmol/L NaCl, 5% glycerol, 0.02% DDM, 250 mmol/L imidazole. Subsequently, the eluate was applied to a HiTrap Q ion exchange column (GE Healthcare), equilibrated with 20 mmol/L Tris•HCl pH 7.4, 5% glycerol, 0.02% DDM. The flow through containing the majority of FlaK, was concentrated and finally applied to a HiLoad Superdex 200 column (GE Healthcare) equilibrated with 20 mmol/L Tris•HCl pH 7.4, 150 mmol/L NaCl, 0.02% DDM.

### Expression and purification of FlaB2

FlaB2 was expressed in *E. coli* Tuner (*DE3*). Cells were grown at 37°C in NZA medium, induced with 1 mmol/L IPTG and harvested after 4 h by centrifugation at 4000 g for 20 min. Membrane fractions were collected after centrifugation at 100,000 g for 1 h, resuspended in a buffer containing 50 mmol/L Tris•HCl pH 7.4, 150 mmol/L NaCl, and solubilized with 1% sodium dodecyl sulfate (SDS). The protein was purified at room temperature using a HisTrap FF Crude affinity column (GE Healthcare) and eluted with 50 mmol/L Tris•HCl pH 7.4, 150 mmol/L NaCl, 250 mmol/L imidazole, 1% SDS. Purified FlaB2 was precipitated with acetone to remove the SDS prior to its use in the activity assay.

### In vitro activity assay using purified FlaB2 and FlaK

FlaB2 was resuspended in 50 mmol/L Tris•HCl pH 7.4, 150 mmol/L NaCl at a final concentration of 9 *μ*mol/L. The assay was performed in 50 mmol/L Tris•HCl pH 7.4, 150 mmol/L NaCl, 0.5% Triton X‐100 using 0.9 *μ*mol/L purified FlaK, and incubated at 37°C for 1 h. The reaction was stopped after the addition of 2× sample buffer (0.15 mol/L Tris•HCl pH 6.8, 1.2% SDS, 30% glycerol, 15% mercaptoethanol, and a small amount of bromophenol blue) and incubation at 95°C for 5 min. Samples were analyzed by western blot.

### Synthesis of KSKKG‐AMC

The side chain protected peptide Ac‐Lys(Boc)Ser(tBu)Lys(Boc)Lys(Boc)‐OH was manually synthesized on Lys(Boc)‐2‐chlorotrityl‐chloride resin (0.94 mmol g^−1^, IRIS Biotech GmbH) using a standard 9H‐fluoren‐9‐ylmethoxycarbonyl (Fmoc) solid phase peptide synthesis protocol. These Fmoc amino acids (four equiv) were activated with O‐(benzotriazol‐1‐yl)‐N,N,N’,N’‐tetramethyluronium hexafluorophosphate (HBTU) (four equiv) and 1‐Hydroxybenzotriazol (HOBt) (4 equiv) in the presence of diisopropylethylamine (DIPEA) (eight equiv) and coupled 2 × for 0.5 h. After washing with N,N‐dimethylformamide (DMF), Fmoc‐protection was removed with 20% piperidine in DMF for 5 and 15 min, respectively. Acetylation of the peptide at the N‐terminus was achieved with acetanhydride, N–methylimidazole, and DMF (1:2:3, v/v/v) for 30 min. After washing with DMF and dichloromethane (DCM), the peptide was cleaved from the resin with 1% trifluoroacetic acid (TFA) in DCM for 3 h. The crude side chain‐protected peptide was then precipitated in ice‐cold diethyl ether, washed three times with diethyl ether, dissolved in 80% tert‐butanol, and finally lyophilized. This peptide was coupled with Gly‐AMC in solution using fluoro‐N,N,N′,N′‐tetramethylformamidinium hexafluorophosphate (TFFH). For this reason, the peptide was dissolved in dimethylsulfoxide (DMSO) and incubated with one equiv. TFFH and two equiv. DIPEA for 12 min. Afterwards, the Gly‐AMC, dissolved in DMSO, was coupled to the peptide overnight. After lyophilization, the side‐chain protecting groups were removed by treatment with TFA and DCM (1:1, v/v) for 1 h and the solvent was removed by evaporation. The resulting peptide was dissolved in 80% tert‐butanol and lyophilized. Finally, the peptide was purified using a semipreparative reversed‐phase HPLC (LC 8A, Shimadzu). Details are given in the Data S1. The identity and purity were controlled by analytical reversed‐ phase HPLC (LC 10AT, Shimadzu, Duisburg, Germany) and MALDI‐ TOF mass spectrometry (Ultraflex II, Bruker Daltonics, Bremen, Germany) (MS calc. 745.87, found 746 (M+H)^+^, analytical HPLC: elution at 20.1 min).

### Synthesis of Abz‐GKSKKGASGIG‐Phe(4‐NO_2_)‐NH_2_×4 TFA

The peptide was prepared by automated solid phase peptide synthesis in a Syro 2000 syntheziser (MultiSynTech GmbH, Witten, Germany) on 100 mg Rink‐amide resin (Novabiochem) using a standard Fmoc‐protocol with double couplings in DMF (fourfold excess of Fmoc amino acids, HOBt, and HBTU in the presence of eight equiv. DIPEA (diisopropylethylamine)), whereby Boc‐2‐Abz‐OH was used as final residue. The Fmoc group was always removed with 20% piperidine in DMF (5 and 20 min). The peptide was cleaved and deprotected by treatment of the resin with a cocktail of TFA/triisopropylsilane/water (95/2.5/2.5, v/v/v) over a period of 2 h at room temperature. The peptide was precipitated in diethyl ether and purified by preparative HPLC (MS calc. 1298.67, found 650.64 (M+2H)^2+^/2, analytical HPLC: elution at 17.97 min). Details are given in the Data S1.

### Fluorescence‐based activity assay

To analyze whether KSKKG‐AMC or Abz‐KSKKGASGIG‐Phe(4‐NO_2_)‐amide are cleaved by FlaK, different concentrations of the substrate were incubated in the dark with 0.5 *μ*g enzyme in 50 mmol/L Tris•HCl, pH 7.4, 150 mmol/L NaCl, 0.5% Triton X‐100, at 37°C for 1 h. The reaction was monitored by measuring the fluorescence at 460 nm after excitation at 380 nm for free AMC and at 420 nm after excitation at 320 nm for Abz (RF‐5301 PC spectrofluorometer, SHIMADZU and spectrofluorometer FP‐6500, JACSO). As control, the peptides were incubated with pronase and aminopeptidase to cleave KSKKG‐AMC or with pronase in the case of Abz‐KSKKGASGIG‐Phe(4‐NO_2_)‐amide. Reaction was performed in 50 mmol/L Tris•HCl, pH 7.4, 150 mmol/L NaCl at 37°C for 1 h.

### Synthesis of inhibitors

#### 3‐Bromo‐thiophene‐2‐sulfonic acid (4‐trifluoromethyl‐benzyl)‐[(3S,4S)‐4‐(4‐trifluoromethyl‐benzylamino)‐pyrrolidin‐3‐yl]‐amide (9)

To a solution of compound **6** (161 mg, 0.22 mmol) in dry DCM (2 mL), HCl (2 mL, 2.0 M in Et_2_O) was added and the reaction mixture was stirred for 24 h at RT. The solvent was removed under reduced pressure and the remaining oily residue was purified via flash chromatography (DCM/MeOH/NH_3(MeOH)_: 85:15:0.1) giving rise to **9** (128 mg, 92%) as a light brown solid.

^1^H‐NMR (400 MHz, CDCl_3_): *δ* = 7.58–7.50 (m, 6H), 7.51 (d, ^*3*^ *J* = 5.3 Hz, 1H), 7.27 (d, ^*3*^ *J* = 8.0 Hz, 2H), 7.08 (d, ^*3*^ *J* = 5.3 Hz, 1H), 5.00 (d, ^*2*^ *J* = 16.7 Hz 1H), 4.55 (d, ^*2*^ *J* = 16.7 Hz 1H), 4.46 (sm, 1H), 3.49–3.37 (m, 3H), 3.30–2.98 (m, 4H), 2.73 (brs, 1H); ^13^C‐NMR (125 MHz, DMSO‐*d*_*6*_, 30.0°C): *δ* = 145.6, 143.8, 135.9, 135.1, 133.7, 128.9, 128.6 (q, ^*2*^ *J*_*C,F*_ = 31.8 Hz), 128.2, 125.5 (q, ^*3*^ *J*_*C,F*_ = 3.9 Hz), 124.7 (q, ^*1*^ *J*_*C,F*_ = 272.6 Hz), 114.4, 61.8, 58.8, 50.8, 48.4, 44.1; MS(ES+): *m/z* (%): 644 (100, [*M* ^81Br^+H]^+^), 642 (100, [*M* ^79Br^+H]^+^); HRMS (ES+) calcd for C_24_H_23_BrF_6_N_3_O_2_S_2_: 642.0314, found: 642.0309; Anal. calcd for C_24_H_22_BrF_6_N_3_O_2_S_2_*1.5H_2_O (%): C: 43.06, H: 3.76, N: 6.28, found: C: 43.01, H: 3.84, N: 6.37. For the respective ^1^H‐NMR spectrum, please refer to the Data S1.

#### 3‐*p*‐Tolyl‐thiophene‐2‐sulfonic acid (4‐trifluoromethyl‐benzyl)‐[(3S,4S)‐4‐(4‐trifluoromethyl‐benzylamino)‐pyrrolidin‐3‐yl]‐amide (10)

To a solution of **9** (150 mg, 0.23 mmol) in toluene (4 mL), *p*‐tolylboronic acid (88 mg, 0.64 mmol), Cs_2_CO_3_ (208 mg, 0.94 mmol), *rac*‐BINAP (44 mg, 0.07 mmol), and Pd‐(II)‐acetate (12 mg, 0.05 mmol) were added. The reaction mixture was stirred for 5 min at RT and then for one hour utilizing microwave irradiation (ramp: 5.0 min, hold: 60 min, temperature 150°C, p_max_: 250 psi, power: 200 W). After filtration of the reaction mixture, the organic layer was washed with brine and concentrated in vacuo. **10** (53 mg, 39%) was obtained after flash column chromatography (DCM/MeOH/NH_3(MeOH)_: 90:10:0.1) of the remaining residue as brownish solid.

^1^H‐NMR (400 MHz, MeOH‐*d*_*4*_): *δ* = 7.79 (d, ^*3*^ *J* = 5.0 Hz, 1H), 7.55 (d, ^*3*^ *J* = 8.0 Hz, 2H), 7.47 (d, ^*3*^ *J* = 7.8 Hz, 2H), 7.46 (d, ^*3*^ *J* = 8.0 Hz, 2H), 7.33 (d, ^*3*^ *J* = 8.0 Hz, 2H), 7.26 (d, ^*3*^ *J* = 8.5 Hz, 2H), 7.25 (d, ^*3*^ *J* = 7.8 Hz, 2H), 7.14 (d, ^*3*^ *J* = 5.3 Hz, 1H), 4.10 (d, ^*2*^ *J *= 17.2 Hz, 1H), 4.05–3.96 (m, 1H), 3.85 (d, ^*2*^ *J* = 16.9 Hz, 1H), 3.53 (d, ^*2*^ *J* = 13.7 Hz, 1H), 3.43 (d, ^*2*^ *J *= 13.7 Hz, 1H), 2.84–2.77 (m, 2H), 2.62–2.54 (m, 1H), 2.49–2.39 (m, 2H), 2.36 (s, 3H); MS(ES+): *m/z* (%): 654 (100, [*M*+H]^+^); HRMS (ES+) calcd for C_31_H_30_F_6_N_3_O_2_S_2_: 654.1678, found: 654.1664; Anal. calcd for C_31_H_29_F_6_N_3_O_2_S_2_ (%) * 0.2 H_2_O: C: 56.65, H: 4.51, N: 6.39, found: C: 56.99, H: 4.90, N: 6.13. For the respective ^1^H‐NMR spectrum, please refer to the Data S1.

### Screening of inhibitors

To identify possible inhibitors of FlaK, the in vitro activity assay using purified FlaK and FlaB2 was performed as described above. If applicable, 3.6 *μ*mol/L–3.6 mmol/L of synthesized inhibitor dissolved in DMSO was preincubated at 37°C with FlaK. In case of pepstatin, acetyl‐pepstatin, pepstatin A methyl ester, *γ*‐secretase‐inhibitor II, *γ*‐secretase‐inhibitor X, and *γ*‐secretase‐ inhibitor XXI 14 nmol/L purified FlaK was incubated in the presence of 18 *μ*mol/L–200 *μ*mol/L inhibitor. FlaB2 was added 2 h after preincubation of FlaK with the inhibitors.

## Results

### Purification of FlaK and preflagellin peptidase assay

**Figure 1:**
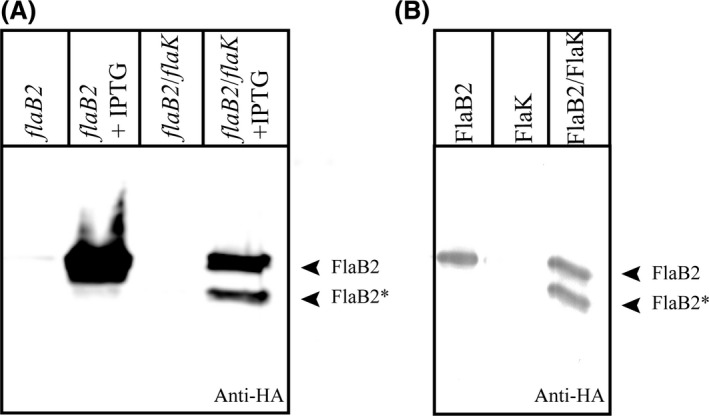
Activity assay of FlaK. (A) In vivo activity assay, showing processing of FlaB2 by FlaK upon co‐expression of the protease and its substrate in *E. coli*, indicated by an additional band at lower molecular weight. (B) After purification of both proteins, FlaB2 is cleaved in vitro by FlaK in the presence of Triton X‐100. Bands representing FlaB2 and the processed FlaB2* are indicated by arrows.

Screening for inhibitors requires the availability of a robust activity assay based on purified components. To clarify whether FlaK of *M. maripaludis* is actively expressed in *E. coli* TUNER (DE3), we co‐expressed the enzyme with its substrate FlaB2. After induction with IPTG, both proteins should be expressed leading to a cleavage of the signal peptide of FlaB2 by FlaK. Indeed, using western blot analysis against FlaB2, two bands representing the immature preflagellin FlaB2 and the mature flagellin FlaB2* were observed. In contrast, the expression of FlaB2 alone resulted in only one band of ~ 25 kDa (Fig. 1A). This shows that the heterologously expressed FlaK is proteolytically active and excludes the presence of other peptidases capable of FlaB2 processing within the expression host. Based on this in vivo activity assay, we developed an in vitro assay with isolated and purified proteins. FlaK was solubilized from the membrane using the standard detergent DDM and purified to homogeneity by column chromatographic techniques. FlaB2, in contrast, could only be solubilized with the denaturing detergent SDS. Thus, FlaB2 was purified under denaturing conditions and its employment in the activity assay required the removal of SDS using acetone precipitation. Upon incubation with purified FlaK, this FlaB2 preparation was cleaved to FlaB2* resulting in the expected band with lower molecular weight upon analysis of the reaction by western blot (Fig. 1B). Interestingly, this reaction occurred in the detergent solubilized state without the addition of any lipid, showing that no membrane is required for the proteolytic reaction. Unfortunately, we did not observe complete conversion of FlaB2 to FlaB2* upon extended treatment with active FlaK (data not shown), indicating that only about 50% of the substrate is in a state or conformation that can be processed or that FlaK is inhibited by the product FlaB2*. Nevertheless, this band shift assay is highly useful and is the basis for the development of specific FlaK inhibitors.

### Fluorogenic substrates of the type X_n_‐AMC are not cleaved by FlaK

In an attempt to develop a fluorogenic FlaK activity assay, we tested several potential substrates. Ng et al. showed that at least four amino acids before the scissile peptide bond (G12‐↓‐A13, where “↓” represents the scissile peptide bond) are necessary for an effective cleavage of FlaB2 by FlaK (Ng et al. 2009), which is in line with our results employing different short peptidic substrates of the general structure X_n_‐AMC (X: any amino acid, *n* = 2–5; data not shown). The longest substrate tested was the peptide KSKKG‐AMC. None of these potential substrates was cleaved by FlaK. Only respective control reactions with aminopeptidase and pronase showed an increase in fluorescence due to the release of free AMC (data not shown).

### Abz‐GKSKKGASGIG‐Phe(4‐NO_2_)‐amide is not cleaved by FlaK

As aspartic proteases typically recognize also amino acids C‐terminal to the cleavage site, we synthesized a peptide with five additional amino acids C‐terminal to the scissile peptide bond. To quantify the reaction, the fluorophore Abz was coupled to the N‐terminus and the quencher Phe(4‐NO_2_)‐NH_2_ was coupled at the C‐terminus. Also in this assay, no cleavage by FlaK could be seen, whereas the respective control reactions with pronase did show the expected increase in fluorescence (data not shown). Therefore, FlaK seems to recognize more than the residues directly encompassing the scissile peptide bond. Subsequently, we used the band shift activity assay based on purified FlaB2 for the screening of various inhibitors.

### Pepstatin and typical γ‐secretase inhibitors do not inhibit FlaK

**Figure 2:**
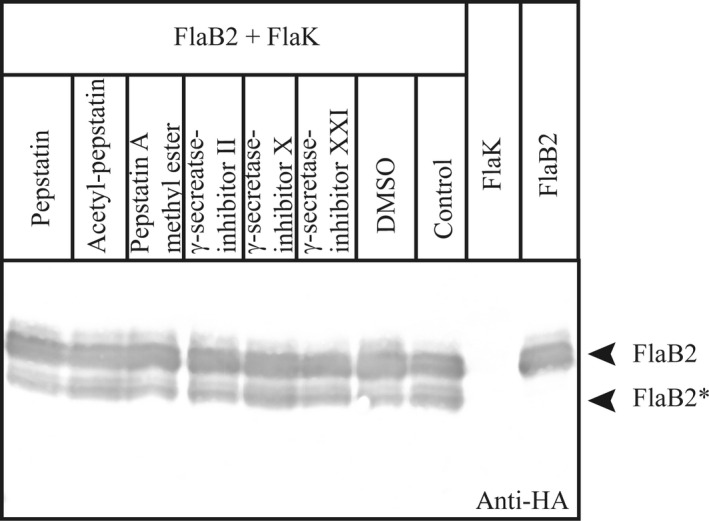
In vitro activity assay in the presence of aspartic protease and *γ*‐secretase inhibitors. 14 nM of purified FlaK was incubated in the presence of 18 *μ*M of different known aspartic protease and *γ*‐secretase inhibitors and mixed with purified FlaB2. FlaB2 or FlaK alone as well as a mixture of both proteins in the presence of the inhibitor solvent dimethylsulfoxide served as controls. The substrate and the processed FlaB2* are indicated by arrows.

FlaK contains two catalytically active aspartates. To determine, whether FlaK can be inhibited by classical aspartic protease inhibitors, we performed the in vitro activity assay with purified components in the presence of various protease inhibitors (Fig. 2). These inhibitors were dissolved in DMSO that alone does not inhibit the cleavage reaction. Nearly, all known aspartic proteases are inhibited by pepstatin, a naturally occurring hexapeptide (Morishima et al. 1970). Interestingly, this aspartic protease inhibitor and also acetyl‐pepstatin did not inhibit FlaK. Even a 13000‐fold molar excess did not prevent processing. We also tested the known *γ*–secretase inhibitors pepstatin A methyl ester, *γ*‐secretase‐inhibitor II, *γ*‐secretase‐inhibitor X, and *γ*‐secretase‐inhibitor XXI, and found that also none of them inhibited the cleavage of FlaB2 by FlaK (Fig. 2). Accordingly, FlaK can neither be inhibited by the classical aspartic protease inhibitors Pepstatin and Acetyl‐Pepstatin nor by typical *γ*‐secretase inhibitors.

### FlaK is inhibited by **9**

**Figure 3:**
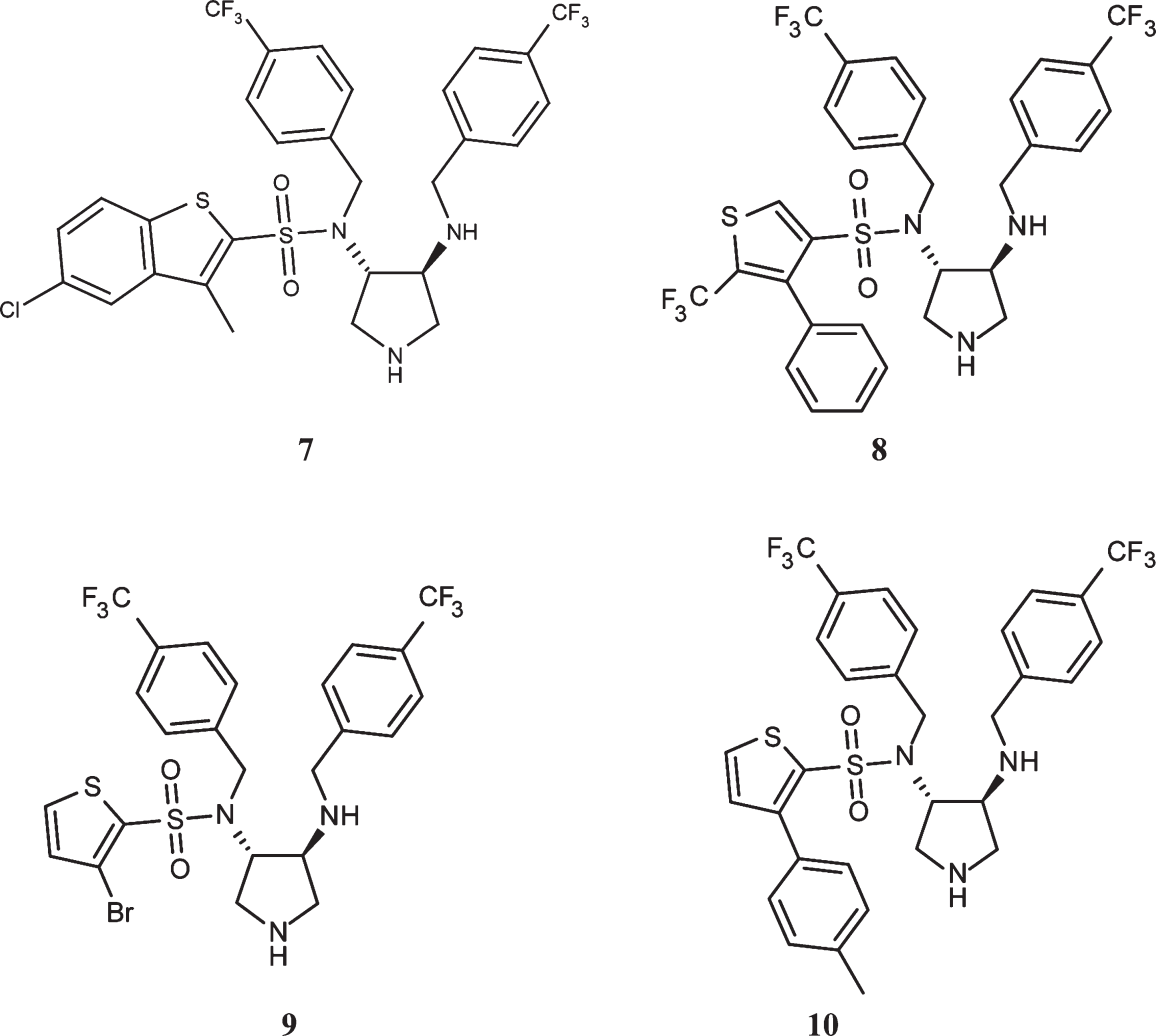
Chemical formulae of **7**,** 8**,** 9,** and **10**.

**Figure 4:**
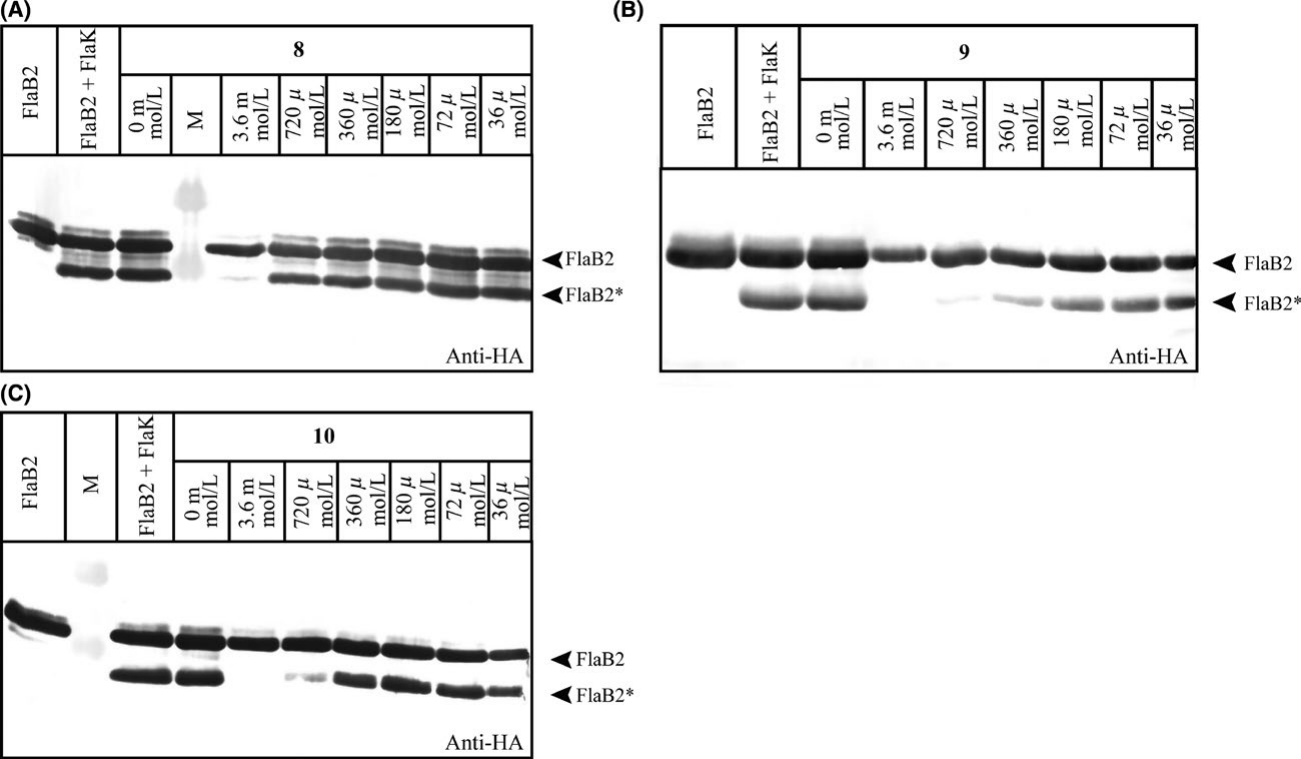
In vitro activity assay in the presence of the synthesized inhibitors **8** (A), **9** (B), and **10** (C). Purified FlaK was incubated with 36 *μ*mol/L‐3.6 mM of potential inhibitors in the presence of Triton X‐100. As control, untreated FlaB2 and FlaK were applied. FlaB2 and the processed FlaB2* are indicated by arrows.

To identify a possible inhibitor of FlaK, we used the developed band shift assay and screened 32 selected compounds of a small in‐house library of totally 110 structurally diverse aspartic protease inhibitors that had been initially designed to target either pepsin‐like (family A1) or viral aspartic proteases (family A2) (Blum et al. 2008a,b; Luksch et al. 2008, 2010; Rawlings et al. 2014). The identification of such an inhibitor stabilizing the active site of FlaK would be extremely useful for X‐ray crystallographic studies of the enzyme in order to catch and to preserve the active site in a catalytically competent conformation. Most of these compounds did not inhibit FlaK, but we identified one compound, **8** (Fig. 3), that significantly reduced the processing of FlaB2 by FlaK, resulting in almost complete inhibition of cleavage at concentrations of 3.6 mmol/L (Fig. 4A). Interestingly, though the structurally related compound **7** was shown to be inactive. Thus, we initially focused in a ligand‐based approach on the variation of the sulfonyl substituent to deduce first structure–activity relationships and synthesized a small series of 12 pyrrolidine‐based derivatives. As most active compounds, we identified **9** and **10** (Fig. 3 and 4). Although cleavage of FlaB2 is completely prevented only in the presence of 3.6 mmol/L **10**,** 9** completely inhibits processing by FlaK already at concentrations of 720 *μ*mol/L (Fig. 4B) and thus represents the most promising compound at present.

### Synthesis of FlaK‐inhibitors

**Figure 5:**
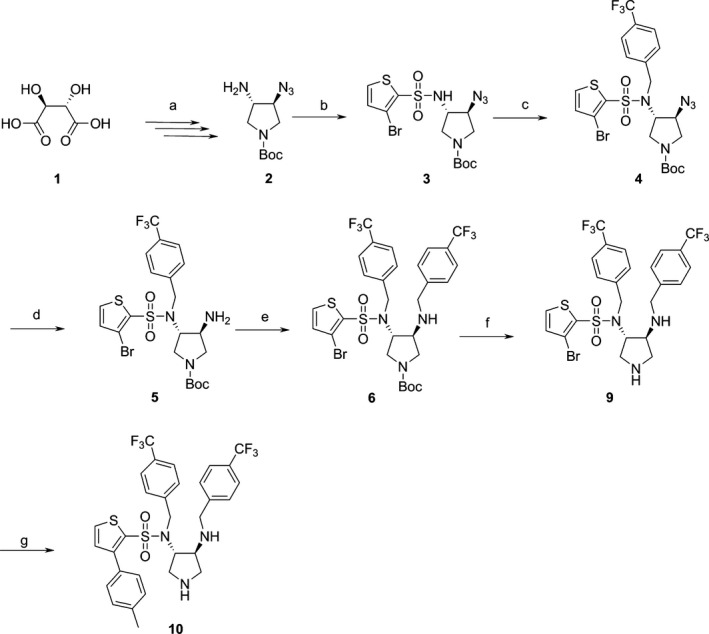
Schematic representation of the Synthesis of **9** and **10**. Reagents and conditions: (a) (Blum et al. 2008a; Klee 2013) (b) 4‐(dimethylamino)pyridine, 3‐bromo‐thiophene‐2‐sulfonyl chloride, pyridine, 0°C → RT, 48 h, 91% (c) NaH, 4‐(trifluoromethyl)‐benzyl bromide, N,N‐dimethylformamide, RT, 20 h, 91%; (d) i) TPP, dichloromethane (DCM), −10°C, 1.5 h, RT, ii) RT, 3 h, iii) NH_3_/MeOH, 50°C, 48 h; (e) i) molecular sieve, 4‐(trifluoromethyl)‐benzaldehyde, MeOH, RT, 24 h, ii) NaBH_4_, 0°C, 4 h, 69% over two steps; (f) 2 M HCl/Et_2_O, DCM, 24 h, 92%; (g) *p*‐tolylboronic acid, Cs_2_ CO_3_, *rac*‐BINAP, Pd‐(II)‐acetate, toluene, microwave irradiation (200 W), 150°C, 1 h, 39%.

The stereoselective synthesis of the pyrrolidine core‐structure is achieved via a six‐step sequence starting from commercially available D‐(‐)‐tartaric acid **1** giving rise to the monoazide **2** (Blum et al. 2008a; Klee 2013) (Fig. 5). Sulfonylation of **2** with 3‐bromo‐thiophene‐2‐sulfonyl chloride to **3**, followed by alkylation of the secondary sulfonamide with 4‐CF_3_‐benzylbromide yields **4**. Subsequent reduction in its azide substituent via a Staudinger reduction to amine **5**, followed by reductive amination employing 4‐CF_3_‐benzaldehyde yielding **6** and final deprotection of the pyrrolidine nitrogen gives rise to the key intermediate **9**. Suzuki coupling of **9** utilizing *p*‐tolylboronic acid yield **10**.

## Discussion

FlaK of *M. maripaludis* is regarded as an aspartic protease showing major differences to the classical pepsin‐like family members. To characterize this novel family of proteases, we developed an in vivo and in vitro activity assay for FlaK of *M. maripaludis*. Since this enzyme was able to process FlaB2 of *M. voltae* (Correia and Jarrell 2000), we expected that FlaK should also cleave the natural substrate FlaB2 of *M. maripaludis*. Co‐expression of FlaK and its substrate FlaB2 in *E. coli* clearly showed processing of this preflagellin and indicated the expression of proteolytically active FlaK.

To use isolated components for an activity assay, we independently purified FlaK and FlaB2. Although FlaK could be solubilized by DDM, solubilization of FlaB2 was only achieved with the denaturing detergent SDS. Since the processed substrate can be solubilized with nondenaturing detergents like DDM or N,N‐dimethyl‐n‐dodecylamine N‐oxide (I. Coburger, unpublished), the peptide sequence preceding the cleavage site in FlaB2 seems to tightly retain the protein in the membrane. Interestingly, we could also show that the denatured and precipitated FlaB2 is processed by purified FlaK in the presence of Triton‐X‐100 only and thus in the absence of any lipid. In contrast, for the TFPPs, for PilD of *Pseudomonas aeruginosa*, and for VcpD of *Vibrio cholerae*, cardiolipin was shown to be necessary for processing of their substrates (Nunn and Lory 1991) (and I. Coburger, unpublished). Thus, a stabilization of the active conformation (McAuley et al. 1999) similar to the TFPPs is apparently not necessary for FlaK, and a less complex structure and reaction mechanism can be expected for the latter enzyme.

In order to understand the substrate recognition requirements of FlaK, we used different synthesized peptides as potential substrates and analyzed, if they are processed by this intramembrane aspartic protease. Ng et al. demonstrated that signal peptides with a length of five amino acids are processed by FlaK (Ng et al. 2009). In addition, it could be shown that a glycine at position P3′ in FlaB2 is highly conserved and necessary for processing (Thomas et al. 2001). Neither the synthesized peptide KSKKG‐↓‐AMC, containing the fluorophore in the P1′ position nor the internally quenched fluorogenic substrate Abz‐GKSKKG‐↓‐ASGIG‐Phe(4‐NO_2_)‐NH_2_, providing five native amino acid residues on either side of the scissile peptide bond, could, however, serve as substrates for FlaK. On the other hand, FlaK was highly active in a band shift assay. Thus, we conclude that it seems to recognize residues or molecular features outside of the immediate environment of the scissile peptide bond, likely the transmembrane helix of its substrates. Interestingly, Ng et al. (Ng et al. 2009) used peptides as substrate containing 19 amino acids C‐terminal to the cleavage site. This corresponds roughly to the number of amino acids required to build up one transmembrane helix. Another reason might be that parts of the used peptides such as the C‐terminal Nitro‐Phe moiety, AMC or N‐terminal Abz‐residue sterically avoided binding of the peptide to FlaK.

Our analysis of different known inhibitors employing the developed in vitro band shift activity assay of FlaK clearly showed that this enzyme does not belong to the family of prototypical aspartic proteases. Although pepstatin is an universal inhibitor for all types of aspartic proteases (Marciniszyn et al. 1976), FlaB2 is processed in the presence of this compound. TFPPs are also insensitive to pepstatin and therefore referred to as nonpepsin‐like acid proteases (Lapointe and Taylor 2000). Accordingly, the reaction mechanism and the active site of FlaK and TFPPs could be similar. This notion is further supported by an architecture of FlaK that differs from pepsin (Hu et al. 2011) and the presence of the conserved GxGD motif in FlaK and in the TFPPs. These characteristics of FlaK together with a neutral pH optimum indicate also similarities to presenilin, such that a similar reaction mechanism of all three proteases can be suggested. In contrast, pepstatin binds to presenilin and inhibits this protease (Evin et al. 2001; Zhang et al. 2001). In addition, in the presence of different *γ*‐secretase inhibitors FlaK is not inhibited, indicating also differences in the reaction mechanism between FlaK and presenilin. In line with this, the crystal structure of the presenilin‐like I‐CLiP PSH (Li et al. 2013; Dang et al. 2015) shows only little structural similarity to FlaK. Accordingly, FlaK, *γ*‐secretase and the TFPPs must also show certain differences regarding their active site topology and their reaction mechanism and no final conclusion on their detailed structural and mechanistic similarity can be drawn at present.

Since pepstatin, acetyl‐pepstatin as well as *γ*‐secretase inhibitors do not inhibit cleavage of FlaB2, we screened a library of different aspartic protease inhibitors. Subsequently, we synthesized a small series of derivatives of the initial screening hit **8** by utilizing a ligand‐based approach. Among them, **9** was identified and shown to be, to the best of our knowledge, the most active inhibitor for FlaK known so far. The developed activity assay does hereby only allow to characterize the relative inhibitory properties of the identified compounds. For the calculation of typically employed inhibitory characteristics like IC_50_ or K_i_‐values, the development of a more quantitative assay is highly desirable. This could possibly be achieved by employing a fluorogenic assay with a longer peptidic substrate such as the assay described by Ng et al. (Ng et al. 2009) (see above).

The available structure of FlaK shows an inactive conformation (Hu et al. 2011). Our identified inhibitors offer now the opportunity for new crystallization attempts in complex with FlaK in order to obtain a high‐resolution structure of the protease in an catalytically competent state. This would provide not only central insights into the structure–function relationship and inhibition of the archaeal protease FlaK and the formation of archaeal flagella but also largely contribute to our understanding of the similarities and differences between TFPPs and the GxGD‐type I‐CLiP *γ*‐secretase centrally involved in the etiology of the neurodegenerative Alzheimer's disease.

## Conflict of Interest

None declared.

## Supporting information


**Figure S1**. (A) Topology of FlaK and FlaB2. Based on the crystal structure, FlaK consists of six transmembrane helices (Hu et al. 2011), whereas FlaB2 has only a single‐transmembrane helix. The catalytically active aspartates are localized in close proximity to the membrane. The positions of both aspartates as well as the cleavage site of FlaB2 are highlighted. (B) Sequence of FlaB2.
**Data S1.** Supporting Materials and Methods together with supporting data.
