# Supplementary material for: Identification of inhibitors of the transmembrane protease FlaK of Methanococcus maripaludis
Source: Microbiologyopen. 2016 Apr 1;5(4):637–46. doi: 10.1002/mbo3.358 (PMC4985597; doi:10.1002/mbo3.358)
Supplement: Supplementary file 1 — Figure S1. (A) Topology of FlaK and FlaB2. Based on the crystal structure, FlaK consists of six transmembrane helices (Hu et al. 2011), whereas FlaB2 has only a single‐transmembrane helix. The catalytically active aspartates are localized in close proximity to the membrane. The positions of both aspartates as well as the cleavage site of FlaB2 are highlighted. (B) Sequence of FlaB2. Data S1. Supporting Materials and Methods together with supporting data. [file MBO3-5-637-s001.pdf]

Supplementary Figure S1

A

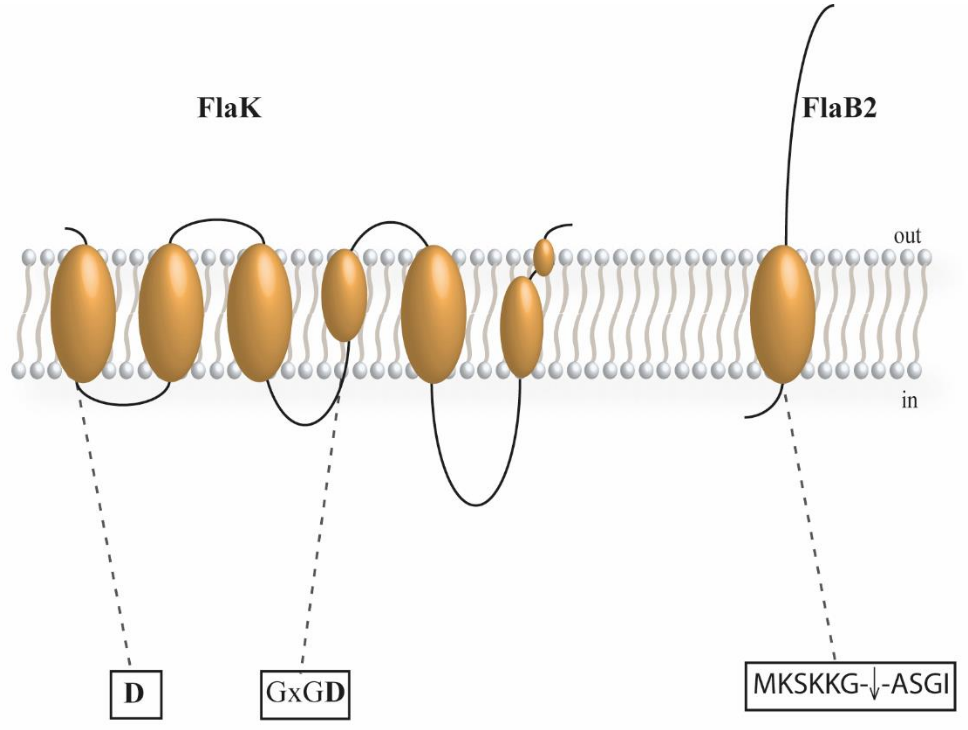

B

MKITEFMKSKKG-↓-ASGIGTLIVFIAMVLVAAVAASVLINTSGFLQQKASTTGKDSTEQV
ASGLQIMGISGYQAGTANANITKLAIYITPNAGSAAIDMNQVVLTLSDGTTKTVTKYDT
TAYTNLTAGGDLYNTTTVNWSKLADTTEFGIVEIQDADLSFTSSAPVINKGDIVAIIVSGV
SFDTRMEISGTVQPEFGAPGVISFTTPSTFTEKVVSLQLVPRGSYPYDVPDYAHHHHHH

(A) Topology of FlaK and FlaB2. Based on the crystal structure FlaK consists of six transmembrane helices (Hu *et al.*, 2011), whereas FlaB2 has only a single transmembrane helix. The catalytically active aspartates are localized in close proximity to the membrane. The positions of both aspartates as well as the cleavage site of FlaB2 are highlighted. (B) Sequence of FlaB2.

Cloning.

M. maripaludis flaB2 was amplified from p945 (personal gift of S.-V. Albers) using the primers 5`-ATATACATATGAAAATAACAGAATTCAT-3´, 5´-TATGTCGACGGCACATCATACGGATAGCTGCCGCGGCACCAGTTGTAATGA AACTACTTTTTCAG-3´ and 5`-TATGTCGACTTAATGGTGATGGTGATGGTGC GCATAATCCGGCACATCATACGGATAGCTGC-3` to insert a HA- and a His-tag for detection as well as purification purposes. The PCR product was cloned into the pIK2 vector resulting in pIK2-flaB2. The expression vector pIK2 was generated from the pVC11 (M. Ehrmann) vector by a method based on the Quikchange mutagenesis Kit of Stratagene. Thereby, a NdeI restriction site was inserted in multiple cloning site and another NdeI restriction site was removed using the primers 5`-GAGGAGAAATTACATATGAGAGGATCTCACC-3`, 5`-GGTGAGATCTCTCATATG TAATTTCTCCTC-3`, 5`-GTGCACCATATCCGGTGTGAAATACCGCACAGATGCGT AAGGAGAAAATACC-3` and 5`-TCACACCGGATATGGTGCACTCTCAGTACAATCT GCTCTGATGCCGCATAG-3`.

HPLC-protocols used during the synthesis of peptidic compounds.

KSKKG-AMC.

The crude peptides were purified by semipreparative reversed-phase HPLC using a Shimadzu LC-8A system equipped with a C18 column (Knauer Eurospher 100). The gradient elution system was 0.1% TFA in water (eluent A) and 0.1% TFA in acetonitrile/water (9:1) (eluent B). The peptides were eluted with a gradient of 30-90% eluent B in 120 min and a flow rate of 10 mL/min. The peaks were detected at 220 nm. Collected fractions were combined, freeze-dried and stored at -20 °C. Peptide analysis: Peptide purity after purification was confirmed with a Shimadzu LC-10AT chromatograph equipped with a Vydac 218TP54 column (C18, 5 mm particle size, 300 Å pore size, 4.6 x 25 mm) and a Vydac 208TP54 column (C8, 5 mm particle

size, 300 Å pore size, 4.6 x 25 mm). The peptides were eluted with a gradient of 0-80 % eluent B in 80 min. The flow rate was 1 mL/min, eluent A was 0.1% TFA in water, and eluent B was 0.1% TFA in acetonitrile; detection was at 220 nm. The molecular weight of crude and purified peptides was confirmed by MALDI-TOF mass spectrometry with Ultraflex II Bruker mass spectrometer (Bruker Daltonics, Bremen, Germany) using alpha-cyano-4-hydroxycinnamic acid as matrix.

Abz-GKSKKGASGIG-Phe(4-NO$_2$)-NH$_2$

The substrate was purified to more than 95% purity (based on the detection at 220 nm) using preparative HPLC (pumps: Varian PrepStar Model 218 gradient system, detector: ProStar Model 320, fraction collector: Varian Model 701) using a C8 column (Nucleodur, 5 µm, 100 Å, 32 x 250 mm, Macherey-Nagel, Düren, Germany) and a linear gradient of acetonitrile containing 0.1 % TFA at a flow rate of 20 ml/min. The peptide was finally obtained as TFA-salt after lyophilisation (Alpha 2-4 Ldplus, Christ, Osterode am Harz, Germany). Analytical HPLC experiments were performed on a Shimadzu LC-10A system (column: Nucleodur C18, 5 µm, 100 Å, 4.6 x 250 mm, Machery-Nagel, Düren, Germany) with a linear gradient of acetonitrile in water containing 0.1 % TFA (10-35 % acetonitrile in 25 min, detection at 220 nm), at a flow rate of 1 ml/min.

Synthesis of inhibitors.

^{1}H NMR spectrum of **9** in $CDCl_3$

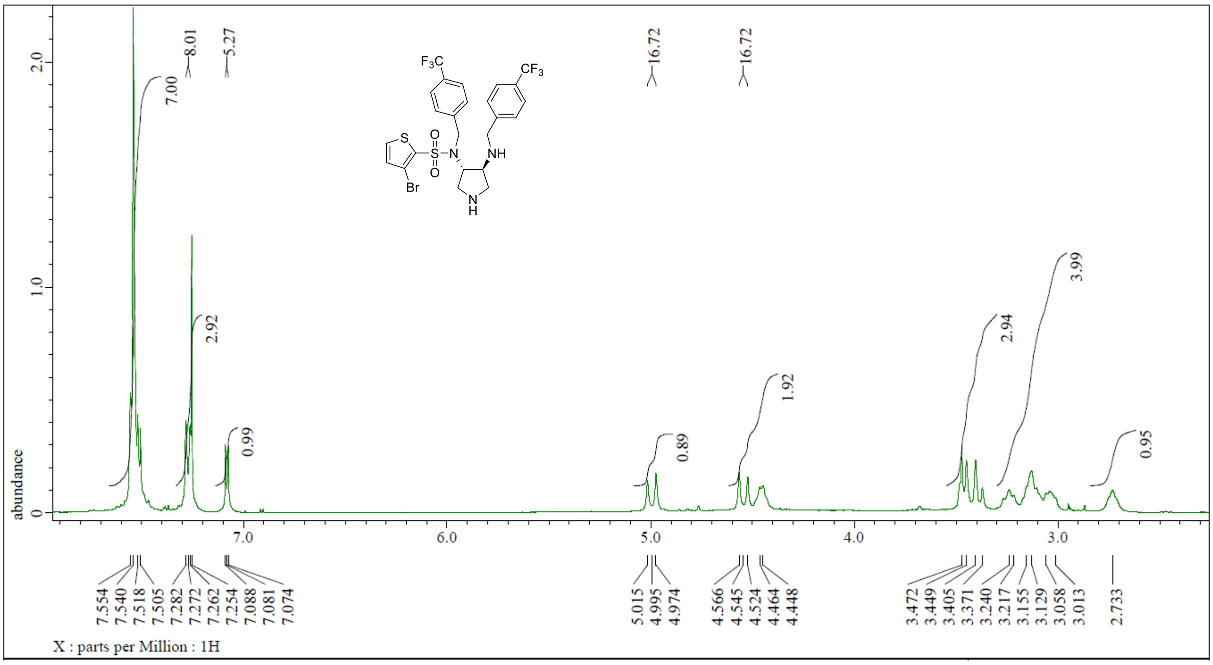

^{1}H NMR spectrum of **10** in CD_3OD

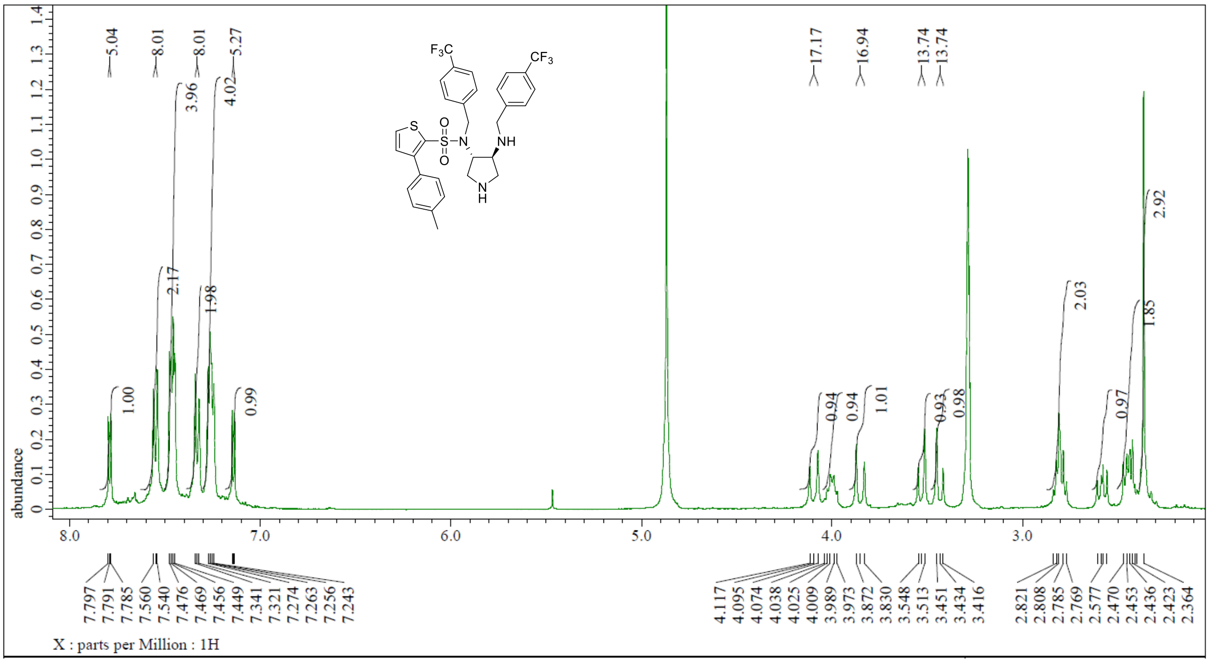

(3*S*,4*S*)-3-Azido-4-(3-bromo-thiophene-2-sulfonylamino)-pyrrolidine-1-carboxylic acid tert-butyl ester (3)

To an icecold solution of **2** (1.00 g, 4.4 mmol) in dry pyridine (50 mL), DMAP (0.27 g, 2.2 mmol) was added and the reaction mixture was stirred for 10 min at 0°C upon which 3-bromo-thiophene-2-sulfonyl chloride (1.73 g, 6.6 mmol), dissolved in dry pyridine (20 mL), was added portion wise. The reaction mixture was allowed to reach RT, stirred for an additional 48 hours and then concentrated in vacuo. Column chromatography (cyclohexane/EtOAc/DCM: 4:4:2) of the remaining residue rendered **3** as yellow-brownish solid (1.71 g, 91%).

^{1}H-NMR (400 MHz, $CDCl_3$): δ = 7.56 (d, ^{3}J = 5.3 Hz, 1H), 7.16 (d, ^{3}J = 5.3 Hz, 1H), 3.97 (sm, 1H), 3.69 (pstd, ^{2}J = 11.7 Hz, ^{3}J = 5.8 Hz, 2H), 3.59 (pstd, ^{2}J = 11.8 Hz, ^{3}J = 5.7 Hz, 1H), 3.42-3.19 (m, 2H), 1.57 (sbr, 1H), 1.44 (s, 9H); ^{13}C-NMR (125 MHz, $CDCl_3$, rotamers): δ = 154.0, 138.7, 133.0, 131.7, 113.9, 80.7, 64.4, 63.4, 57.6, 56.9, 49.8, 49.2, 48.6, 48.3, 28.5; MS(ES+): *m/z* (%): 474 (100, $[M^{79Br}+Na]^+$), 476 (95, $[M^{81Br}+Na]^+$); Anal. calcd for $C_{13}H_{18}BrN_5O_4S_2$ (%): C: 34.52, H: 4.01, N: 15.48, found: C: 34.33, H: 4.04, N: 14.66.

(3*S*,4*S*)-3-Azido-4-[(3-bromo-thiophene-2-sulfonyl)-(4-trifluoromethyl-benzyl)-amino]-pyrrolidine-1-carboxylic acid tert-butyl ester (4)

To a solution of **3** (400 mg, 0.9 mmol) in DMF (10 mL), NaH (49.3 mg, 55% in paraffin, 1.1 mmol) was added and the reaction mixture was stirred for 15 min at RT under an argon atmosphere. After addition of 4-(trifluoromethyl)-benzyl bromide (270 mg, 1.1 mmol), the reaction mixture was stirred for an additional 20 hours, quenched by addition of water and a saturated aqueous Na_2CO_3-solution, extracted three times with EtOAc (50 mL), dried over $MgSO_4$, filtered, and concentrated in vacuo. Flash column chromatography (cyclcohexane/EtOAc: 85:15) of the residue rendered **4** as yellow solid (516 mg, 91%).

^{1}H-NMR (400 MHz, $CDCl_3$): δ = 7.61 (d, ^{3}J = 8.2 Hz, 2H), 7.56-7.46 (m, 3H), 7.11 (brs, 1H), 5.05-4.30 (m, 2H), 3.95-3.77 (sm, 1H), 3.67-3.40 (m, 2H), 3.36-3.32 (m, 1H), 3.22-2.95 (m, 2H), 1.40 (s, 9H); ^{13}C-NMR (125 MHz, $CDCl_3$, rotamers): δ = 153.7, 141.1, 136.2, 133.3, 132.2, 130.5 (q, $^{2}J_{C,F}$ = 32.8 Hz), 127.6, 126.0 (q, $^{3}J_{C,F}$ = 3.9 Hz), 124.0 (q, $^{1}J_{C,F}$ = 272.6 Hz), 114.0, 80.7, 61.4, 61.2, 49.1, 48.5, 44.8, 28.4; MS(ES+): *m/z* (%): 512 (100, $[M^{81Br}$–Boc+H$]^{+}$), 609 (45, $[M^{79Br}$+H$]^{+}$); HRMS (ES+) calcd for $C_{21}H_{23}BrF_3N_5O_4S_2Na$: 632.0219, found: 632.0212; Anal. calcd for $C_{21}H_{23}BrF_3N_5O_4S_2$ (%): C: 41.52, H: 3.80, N: 11.47, found: C: 41.60, H: 3.99, N: 11.28.

(3*S*,4*S*)-3-Amino-4-[(3-bromo-thiophene-2-sulfonyl)-(4-trifluoromethyl-benzyl)-amino]-pyrrolidine-1-carboxylic acid tert-butyl ester (5)

A solution of triphenylphosphine (0.49 g, 1.9 mmol) in DCM (25 mL) was added drop-wise over a period of an hour to a solution of **4** (0.97 g, 1.58 mmol) in dry DCM (50 mL) at -10°C. The reaction mixture was stirred for additional 1.5 hours at -10°C, allowed to reach RT, stirred for an additional 3 hours at RT, and then concentrated under reduced pressure. After addition of an ammonia solution (7 M in MeOH, 35 mL) and water (20 mL) to quench the reaction, the resulting suspension was stirred vigorously for 48 hours at 50°C. Upon addition of toluene (15 mL), the solvent was removed under reduced pressure. Column chromatography (DCM/MeOH/$NH_{3(MeOH)}$: 95:5:0.1) of the residue yielded crude **5**, which was used without further purification.

MS(ES+): *m/z* (%): 585 (15, $[M$+H$]^{+}$).

(3S,4S)-3-[(3-Bromo-thiophene-2-sulfonyl)-(4-trifluoromethyl-benzyl)-amino]-4-(4-trifluoromethyl-benzylamino)-pyrrolidine-1-carboxylic acid tert-butyl ester (6)

To a solution of crude **5** (0.98 g) in dry methanol (30 mL), 0.51 g molecular sieve and 4-(trifluoromethyl)-benzaldehyde (0.45 g, 2.37 mmol) were added and the resulting mixture was

stirred at RT for 24 hours. After cooling to 0°C, $NaBH_4$ (150 mg, 3.48 mmol) was added slowly, the reaction mixture stirred for 4 hours, quenched by the addition of a saturated aqueous $NaHCO_3$-solution, and extracted three times with EtOAc (30 mL). The combined organic layers were dried over $MgSO_4$, filtered, and concentrated in vacuo. Flash column chromatography (cyclohexane/EtOAc: 75:25) of the residue gave rise to **6** (698 mg, 69% over two steps) as colorless crystals.

^{1}H-NMR (400 MHz, $CDCl_3$): δ = 7.58-7.40 (m, 6H), 7.50 (d, ^{3}J = 5.3 Hz, 1H), 7.29 (d, ^{3}J = 8.0 Hz, 2H), 7.08 (d, ^{3}J = 5.3 Hz, 1H), 5.10 (d, ^{2}J=16.9 Hz, 1H), 4.79-4.56 (m, 1H), 4.38 (m, 2H), 3.70-3.50 (m, 1H), 3.46-3.20 (m, 2H), 3.04-2.85 (m, 2H), 2.78 (m, 1H), 1.55 (brs, 1H), 1.39 (s, 9H); MS(ES+): *m/z* (%): 766 (10, [M^{81Br}+Na]$^+$), 742 (100, [M^{79Br}+H]$^+$), 688 (85, [M^{81Br}-*t*Bu+H]$^+$); HRMS (ES+) calcd for $C_{29}H_{31}BrF_6N_3O_4S_2$: 742.0838, found: 742.0832; Anal. calcd for $C_{29}H_{30}BrF_6N_3O_4S_2$ (%): C: 46.90, H: 4.07, N: 5.66, found: C: 47.34, H: 4.51, N: 5.41.
